# Topical Application of Jojoba Oil Suppresses Exercise-Induced Inflammatory Gene Expression in Mouse Skeletal Muscle

**DOI:** 10.3390/biom15101394

**Published:** 2025-09-30

**Authors:** Yutaka Matsumoto, Katsuhiko Suzuki

**Affiliations:** 1 Faculty of Nursing, Tokai University School of Medicine, Isehara 259-1292, Kanagawa, Japan; 2 Faculty of Sport Sciences, Waseda University, Tokorozawa 359-1192, Saitama, Japan

**Keywords:** jojoba oil, sports massage, topical application, transdermal absorption, exercise-induced muscle injury, exercise-induced inflammation, anti-inflammation

## Abstract

*Background and objectives*: Exercise-induced muscle injury, a consequence of intense physical activity, is characterized by subsequent inflammation. Sports massage frequently employs massage oils, such as jojoba (*Simmondsia chinensis* (Link.) C.K.Schneid., Simmondsiaceae) oil, which is recognized for its anti-inflammatory properties. Therefore, this study investigated the potential of jojoba oil to alleviate exercise-induced muscle injury. *Materials and Methods*: Male hairless mice, aged eight weeks, were randomly allocated into one of four groups: a naïve control group, a sedentary group treated with jojoba oil (JO), an exercise group without oil application, and an exercise group treated with jojoba oil (JO + Ex). In the JO and JO + Ex groups, 4 μL of jojoba oil per gram of body weight was applied topically to the dorsal skin of the mice 30 min prior to treadmill exercise. Subsequently, plasma biochemical parameters, gene expression in various tissues, and plasma cytokine levels were evaluated. *Results*: Topical application of jojoba oil did not significantly impact plasma cytokine concentrations. However, it significantly decreased the expression levels of pro-inflammatory cytokines (*Il-1b* and *Il-6* in the soleus muscle; *Il-1b* in the gastrocnemius muscle). *Conclusions*: Our findings suggest that sports massage with jojoba oil may aid in reducing exercise-induced muscle injury and inflammation.

## 1. Introduction

The risk of various diseases, including obesity, hypertension, stroke, coronary heart disease, cancer, type 2 diabetes mellitus, dyslipidemia, osteoporosis, and depression, is reduced by habitual physical activity [1,2,3]. On the other hand, intense physical activity can result in exercise-induced muscle injury and delayed-onset muscle soreness (DOMS) [4]. These conditions are marked by discomfort and secondary inflammation due to the infiltration of leukocytes into the injured muscle tissue [5,6,7,8]. DOMS leads to swelling, muscle weakness, and a decreased range of motion [9,10]. The development of this condition is generally initiated within the first 24 h post-exercise, with its maximum intensity occurring between 24 and 72 h [7]. Consequently, alleviating the symptoms of muscle injury and inflammation can be especially advantageous for those who need to recover quickly [11,12].

Recently, the usefulness of sports massage for reducing exercise-induced inflammation [13] and DOMS [14,15,16], as well as for improving muscle performance, has been reported [9,15]. Massage is believed to promote blood and lymph circulation, enhance muscle metabolism, and relieve fatigue by passively moving and stimulating the muscles. In Europe, where massage originated, various oils including jojoba (*Simmondsia chinensis* (Link.) C.K.Schneid., Simmondsiaceae) oil and sweet almond oil are commonly used in sports massage. These oils reduce friction between the skin and the hands, thereby facilitating smoother massage strokes. Jojoba oil is also employed as a carrier oil for diluting essential oils.

Although massage therapy has been extensively studied, little is known about the biological effects of carrier oils. Some review articles do not specify the types of massage oils used in previous studies [15,17,18,19,20,21], and many researchers and therapists seem to consider their biological effects negligible. However, jojoba oil, commonly used in sports massage, has shown anti-inflammatory effects in models of carrageenin-induced paw edema and croton oil-induced ear edema in rats [22,23]. Its effect on exercise-induced inflammation, however, remains unclear. Therefore, this study focused on the potential of pre-exercise topical application of jojoba oil to prevent inflammation related to muscle injury.

Meanwhile, it is established that lipid consumption rises during endurance exercise [24]. A recent study found that applying jojoba oil topically on mice led to a roughly 20% increase in plasma non-esterified fatty acid (NEFA) levels 30 min later [25]. When skeletal muscles use plasma NEFA as an energy source, it could influence the duration before exhaustion during prolonged exercises. Therefore, this study aimed to investigate whether topical application of jojoba oil prior to treadmill running prevents exercise-induced muscle injury and whether it influences time to exhaustion.

## 2. Materials and Methods

### 2.1. Ethics Statement

All animal experiments were conducted in accordance with the Guidelines for the Care and Use of Laboratory Animals, as stipulated in Act No. 105 and Notification No. 6 of the Government of Japan. All procedures were approved by the Institutional Animal Care and Use Committee of Waseda University (Approval No. 2019-A132).

### 2.2. Animals

Seven-week-old male hairless mice (Hos: HR-1) were procured from Hoshino Laboratory Animals, Inc. (Bando, Japan) via Japan SLC, Inc. (Hamamatsu, Japan) and then acclimated to the breeding facility for at least one week prior to the experiment. At the start of the experiment, all mice were eight weeks old and weighed between 28.2 and 34.8 g (mean ± SD: 31.2 ± 1.4 g).

The mice were housed under specific pathogen-free conditions in a temperature-controlled room (22–24 °C) with 50–60% relative humidity and a 12-h light/dark cycle (lights on at 08:00 and off at 20:00). They were provided with a standard commercial diet (MF; Oriental Yeast, Tokyo, Japan), and water was supplied ad libitum.

### 2.3. Chemicals

Jojoba oil was purchased from Kenso-Igakusha Co., Ltd. (Kai, Japan; Lot No. GL15A). The chemical composition of the jojoba oil used in this study is summarized in Table 1, as provided by the manufacturer’s package insert. The chemical composition of the jojoba oil used in this study was based on an analysis certificate provided by the manufacturer. This analysis was conducted by a third-party accredited institution and did not include detailed profiling of trace components. Unlike conventional vegetable oils, which are primarily composed of triglycerides (TGs), jojoba oil is chemically distinct and consists mainly of wax esters formed from long-chain fatty acids and fatty alcohols.

### 2.4. Experimental Protocol

One week prior to the exhaustive exercise trial, all mice in the exercise groups were acclimated to treadmill running at a speed of 15 m/min on a 0% incline for 10 min using a motorized treadmill. On each experimental day, mice were randomly divided into four groups (*n* = 7 per group): a naïve control group (NA), a sedentary group treated with jojoba oil (JO), an exercise group without oil application (NA + Ex), and an exercise group treated with jojoba oil (JO + Ex).

Based on the findings of previous studies [25,26,27], the mice received a topical application of 4 μL of jojoba oil per gram of body weight to the dorsal skin 30 min before treadmill exercise. Endurance testing was conducted using a motorized treadmill (Natsume Seisakusho Co., Ltd., Tokyo, Japan), and the time to exhaustion was recorded. Mice in the NA + Ex and JO + Ex groups were subjected to a running protocol consisting of 10 m/min for 15 min, followed by 15 m/min and 20 m/min for 15 min each, and then 24 m/min at a constant 7% incline until exhaustion. To control feeding conditions, sedentary mice were fasted during the exercise period. Exhaustion was defined as the point at which the animal was no longer able to maintain running, even after repeated gentle prodding on the back using a silicone rubber spatula.

To minimize observer bias, a single observer, who was blinded to the treatments, determined the endpoint of exhaustion for all time-to-exhaustion tests within the study. Electric shock was not used to avoid causing stress to the mice. This approach represents a humane improvement and reduces the confounding effect of negative reinforcement on blood cytokine levels associated with the use of shock grids [28]. Immediately after exhaustion, blood samples were drawn from the heart under light anesthesia with inhaled isoflurane (Abbott, Tokyo, Japan) and placed in heparinized tubes, after which isolated muscle tissues were immediately frozen in liquid nitrogen. Blood samples were centrifuged at 1500× *g* for 10 min at 4 °C to separate plasma, which was then stored at −80 °C until analysis.

### 2.5. Biochemical Analysis of Plasma

Plasma levels of alanine aminotransferase (ALT), aspartate aminotransferase (AST), alkaline phosphatase (ALP), blood urea nitrogen (BUN), creatinine (CRE), creatine kinase (CK), TG, NEFA, glucose (GLU), and lactic acid (LA) were measured at Kotobiken Medical Laboratories (Tsukuba, Japan). The plasma levels of ALT and AST were assessed by the JSCC transferable method using L-Type ALT J2 and L-Type AST J2, respectively (FUJIFILM Wako Pure Chemical Corporation, Osaka, Japan). The plasma ALP level was measured by the IFCC transferable method using L-Type ALP IFCC (FUJIFILM Wako Pure Chemical Corporation). The plasma BUN level was determined by the urease-GlDH method using L-Type UN·V (FUJIFILM Wako Pure Chemical Corporation). The plasma CRE level was determined by an enzymatic method using Determiner L CRE (Hitachi Chemical Diagnostics Systems Co., Ltd., Tokyo, Japan). The plasma CK level was analyzed by the JSCC transferable method using L-Type CK (FUJIFILM Wako Pure Chemical Corporation). The plasma TG and NEFA levels were quantified by enzymatic method using Determiner C-TG (Hitachi Chemical Diagnostics Systems Co.) and NEFA-HR II (FUJIFILM Wako Pure Chemical Corporation), respectively. The plasma GLU level was measured by the hexokinase-G-6-PDH method using L-Type Glu 2 (FUJIFILM Wako Pure Chemical Corporation) on a BioMajesty^TM^ automated analyzer (JCA-BM9130; JEOL Ltd., Tokyo, Japan). The plasma LA level was quantified by an enzymatic method using Determiner LA (Hitachi Chemical Diagnostics Systems Co.). All measurements, with the exception of GLU, were performed on a BioMajesty^TM^ autoanalyzer (JCA-BM8060; JEOL Ltd.).

### 2.6. RNA Isolation and Gene Expression Analysis Using Real-Time Polymerase Chain Reaction (Real-Time PCR)

Tissue samples were promptly frozen in liquid nitrogen and kept at −80 °C until RNA extraction. To extract total RNA from the soleus muscle, the gastrocnemius muscle, the heart, and the liver, we combined the acid guanidinium thiocyanate-phenol-chloroform method [29,30] with bead homogenization using a Shake Maser Neo (Bio Medical Science, Tokyo, Japan). The purity and quality of the total RNA from each sample were evaluated using a NanoDrop^TM^ 2000c spectrophotometer (Thermo Fisher Scientific, Tewksbury, MA, USA), with the A260/A280 ratio being measured to assess these parameters. Total RNA was reverse-transcribed to cDNA using the High Capacity cDNA Reverse Transcription Kit (Applied Biosystems, Foster City, CA, USA), in accordance with the manufacturer’s instructions. PCR was conducted using the StepOnePlus^TM^ system (Applied Biosystems), with the Fast SYBR^®^ Green Master Mix (Applied Biosystems). The PCR thermal cycling protocol consisted of an initial denaturation step at 95 °C for 10 s, followed by 45 cycles. Each cycle comprised denaturation at 95 °C for 5 s, annealing at 57 °C for 10 s, and extension at 72 °C for 10 s. Relative gene expression levels were normalized to β-actin using the calibration curve method. The specific genes and primers are detailed in Table 2.

### 2.7. Quantitative Analysis of Inflammation-Related Plasma Cytokines

To quantify plasma levels of granulocyte-macrophage colony-stimulating factor (GM-CSF), monocyte chemoattractant protein 1 (MCP-1), interferon (IFN)-β, IFN-γ, interleukin (IL)-1α, IL-1β, IL-6, IL-10, IL-12p70, IL-17A, IL-23, IL-27, and tumor necrosis factor (TNF)-α, we utilized the LEGENDplex^TM^ Mouse Inflammation Panel (Lot No. B298993; Cat No. 740150; BioLegend, San Diego, CA, USA) as per the manufacturer’s instructions. Samples were analyzed on a BD LSRFortessa Special Order Research Product analytical cytometer (Becton Dickinson, San Jose, CA, USA), and the data were processed with the LEGENDplex^TM^ Data Analysis software version 8.0.

### 2.8. Statistical Analysis

Values are presented as mean ± standard error (SE). Normality was confirmed using the Shapiro–Wilk test before comparisons between two groups were conducted using Student’s unpaired *t*-test. For comparisons among four groups, one-way analysis of variance (ANOVA) followed by Tukey’s post hoc test was conducted. Correlations were analyzed using Spearman’s rank correlation coefficient. All statistical analyses were conducted using the SPSS 24.0 software package (IBM Japan Inc., Tokyo, Japan). Statistical significance was defined as *p* < 0.05.

## 3. Results

### 3.1. The Effect of Topical Application of Undiluted Jojoba Oil Before Exercise on Endurance Performance

**Figure 1:**
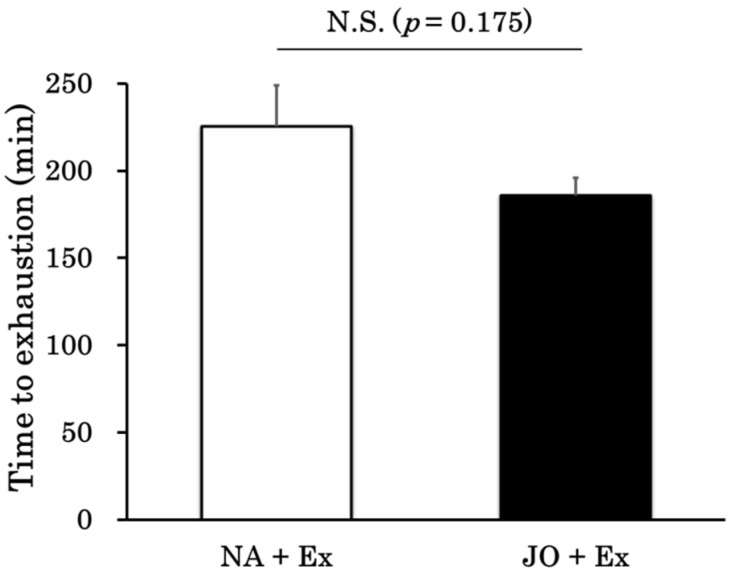
Time to exhaustion in the exercise group without oil application (NA + Ex, *n* = 7) and the exercise group treated with jojoba oil (JO + Ex, *n* = 7). NA + Ex, the exercise group without oil application; JO + Ex, the exercise group treated with jojoba oil. Values are presented as mean ± standard error (SE). Student’s unpaired *t*-test was used for comparisons. N.S., not significant.

The time to exhaustion was 225 ± 23 min in the NA + Ex group and 185 ± 10 min in the JO + Ex group. As shown in Figure 1, there was no statistical difference between the two groups in time to exhaustion (*p* = 0.175).

### 3.2. Plasma Biochemical Analysis

**Figure 2:**
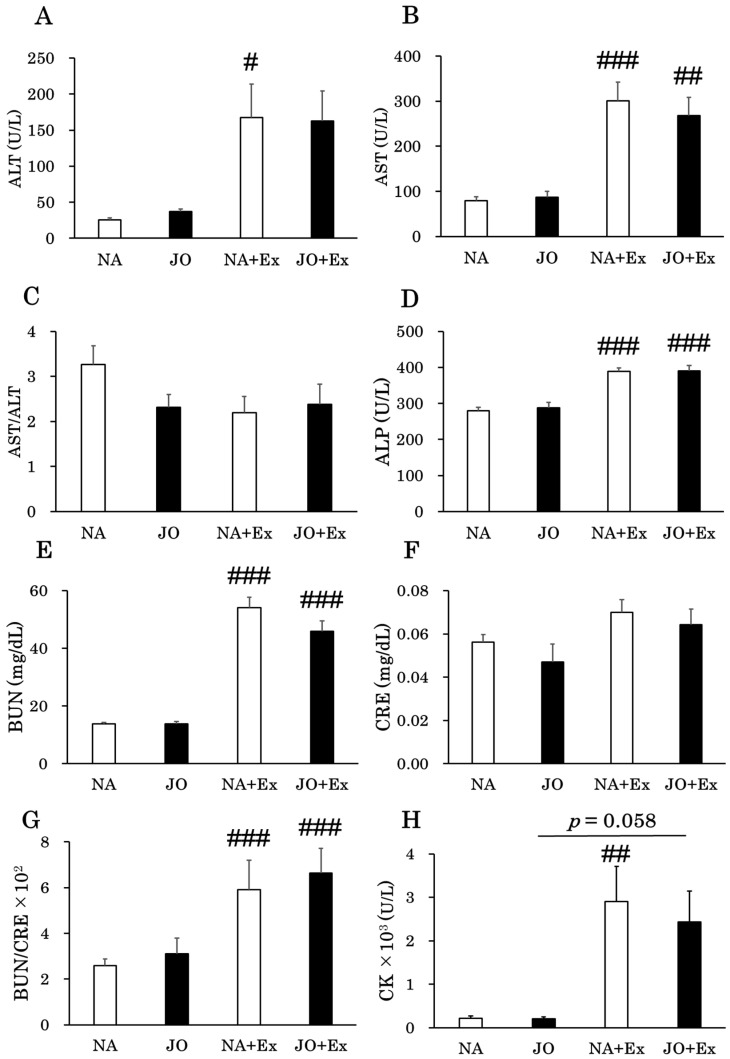

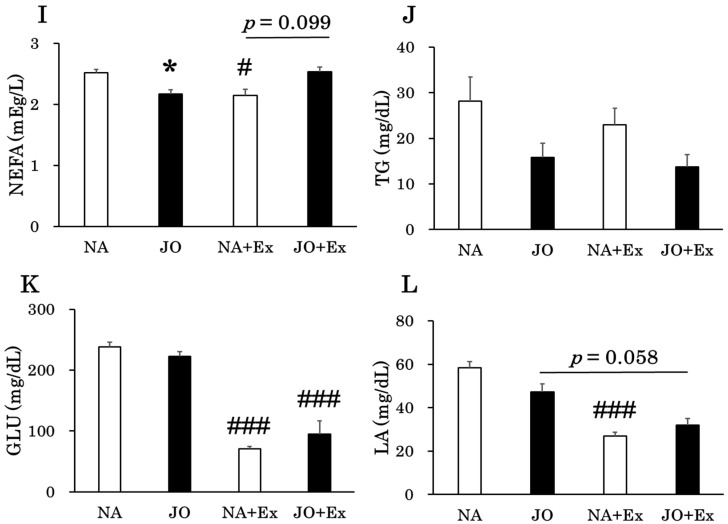
Plasma levels of (**A**) ALT, (**B**) AST, (**C**) AST/ALT, (**D**) ALP, (**E**) BUN, (**F**) CRE, (**G**) BUN/CRE, (**H**) CK, (**I**) NEFA, (**J**) TG, (**K**) GLU, and (**L**) LA. In the exercise groups (NA + Ex, JO + Ex), blood samples were collected from the heart immediately after the point of exhaustion. In the sedentary groups (NA, JO), blood samples were collected at the corresponding time points when the animals in the exercise groups reached exhaustion and were sampled. While mice in the exercise groups were running, those in the sedentary groups were kept in a fasting state to adjust feeding conditions. NA, the naïve control group; JO, the sedentary group treated with jojoba oil; NA + Ex, the exercise group without oil application; JO + Ex, the exercise group treated with jojoba oil. ALT, alanine aminotransferase; AST, aspartate aminotransferase; ALP, alkaline phosphatase; BUN, blood urea nitrogen; CRE, creatinine; CK, creatine kinase; TG, triglyceride; NEFA, non-esterified fatty acids; GLU, glucose; LA, lactic acid. Values are presented as mean ± SE, *n* = 7. Comparisons among four groups were performed using one-way analysis of variance followed by Tukey’s post hoc test. * *p* < 0.05 as compared to the NA; # *p* < 0.05 as compared to the corresponding sedentary group; ## *p* < 0.01 as compared to the corresponding sedentary group; ### *p* < 0.001 as compared to the corresponding sedentary group.

Plasma biochemistry results for all experimental groups, with or without jojoba oil treatment, are shown in Figure 2A–L. The plasma levels of ALT, AST, ALP, BUN, BUN/CRE, and CK were significantly higher in the NA + Ex group than in the NA group, whereas the plasma levels of NEFA, GLU, and LA were significantly lower. Also, the plasma levels of AST, ALP, BUN, BUN/CRE, and CK were elevated in the JO + Ex group compared with the JO group. These differences were statistically significant for AST, ALP, BUN, and BUN/CRE and were borderline significant for CK (*p* = 0.058). A decrease was observed in the plasma levels of GLU and LA. The reduction in GLU levels was statistically significant, while that in LA levels was borderline significant (*p* = 0.058). In the sedentary groups, the only parameter showing a significant difference was NEFA, with levels significantly lower in the JO group than in the NA group. In contrast, no significant changes in any biochemical parameters were observed between the two exercise groups (NA + Ex and JO + Ex) following jojoba oil application.

### 3.3. Effects of Exhaustive Exercise With or Without Topical Application of Jojoba Oil on the Expression Levels of Inflammation-Related Genes in the Soleus Muscle

**Figure 3:**
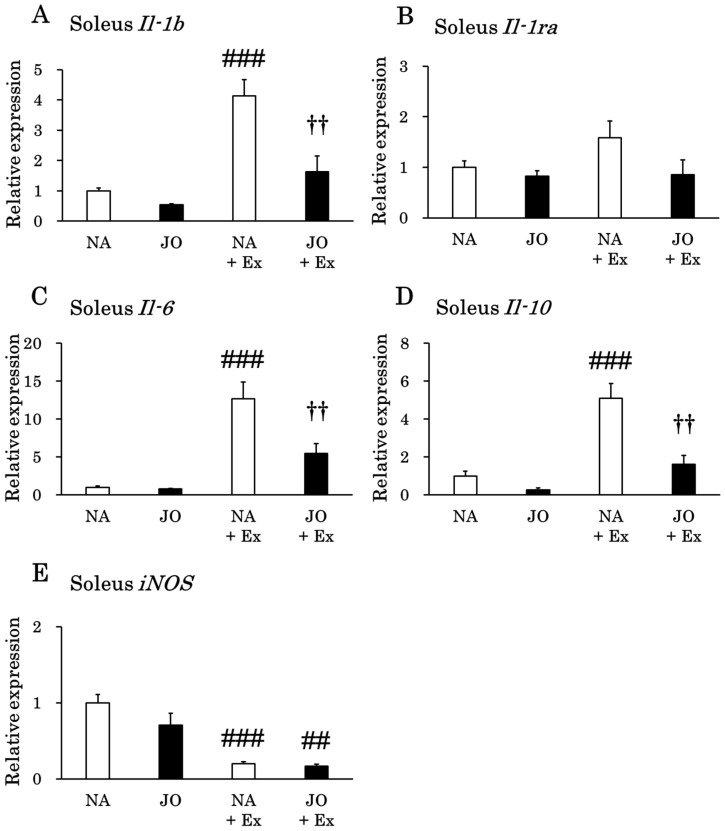
Gene expression levels of (**A**) *Il-1b*, (**B**) *Il-1ra*, (**C**) *Il-6*, (**D**) *Il-10*, and (**E**) *iNOS* in the soleus muscle. NA, the naïve control group; JO, the sedentary group treated with jojoba oil; NA + Ex, the exercise group without oil application; JO + Ex, the exercise group treated with jojoba oil. *Il-1b*, interleukin 1β; *Il-1ra*, interleukin 1 receptor antagonist; *Il-6*, interleukin 6; *Il-10*, interleukin 10; *iNOS*, inducible nitric oxide synthase. Values are presented as mean ± SE, *n* = 7. One-way analysis of variance with Tukey’s post hoc test was employed for comparisons among the four groups. ## *p* < 0.01 as compared to the corresponding sedentary group; ### *p* < 0.001 as compared to the corresponding sedentary group; †† *p* < 0.01 as compared to the NA + Ex group.

This study investigated the anti-inflammatory effect of the topical application of jojoba oil on treadmill-running mice by measuring the expression levels of inflammation-related mRNA in various organs and tissues. In the soleus muscle, the expression levels of *Il-1b*, *Il-6*, and *Il-10* genes were significantly increased by exhaustive exercise (Figure 3). However, the JO + Ex group did not show a significant increase in the expression levels of these genes after treadmill running. In the exercise groups, the gene expression levels of *Il-6* and *Il-10* were significantly lower in the JO + Ex group than in the NA + Ex group. Also, the expression levels of inducible nitric oxide synthase (*iNOS*) were significantly decreased in both exercise groups. However, no significant differences in *Il-1ra* expression levels were observed among the four groups. Additionally, in the comparison between the sedentary groups, the application of jojoba oil did not alter the expression levels of inflammation-related genes.

### 3.4. Effects of Exhaustive Exercise With or Without Topical Application of Jojoba Oil on the Expression Levels of Inflammation-Related Genes in the Gastrocnemius Muscle

**Figure 4:**
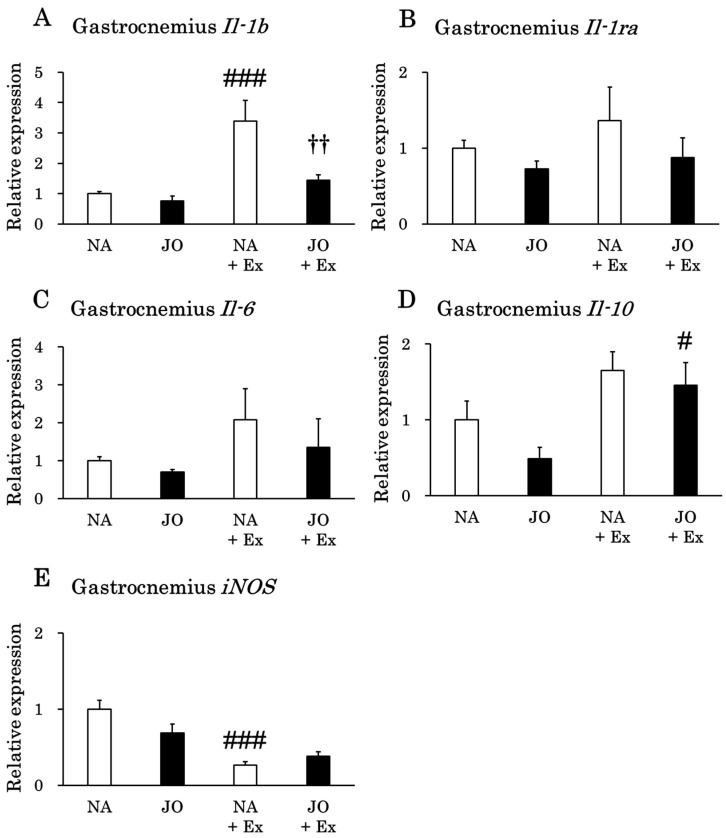
Gene expression levels of (**A**) *Il-1b*, (**B**) *Il-1ra*, (**C**) *Il-6*, (**D**) *Il-10*, and (**E**) *iNOS* in the gastrocnemius muscle. NA, the naïve control group; JO, the sedentary group treated with jojoba oil; NA + Ex, the exercise group without oil application; JO + Ex, the exercise group treated with jojoba oil. *Il-1b*, interleukin 1β; *Il-1ra*, interleukin 1 receptor antagonist; *Il-6*, interleukin 6; *Il-10*, interleukin 10; *iNOS*, inducible nitric oxide synthase. Values are presented as mean ± SE, *n* = 7. Differences among the four groups were analyzed by one-way analysis of variance, with post hoc comparisons conducted using Tukey’s test. # *p* < 0.05 as compared to the corresponding sedentary group; ### *p* < 0.001 as compared to the corresponding sedentary group; †† *p* < 0.01 as compared to the NA + Ex group.

Exhaustive exercise significantly increased the expression level of *Il-1b* in the gastrocnemius muscle (Figure 4). However, this increase was significantly attenuated in the JO + Ex group compared to the NA + Ex group. Conversely, *Il-10* expression levels were significantly elevated in the JO + Ex group by exercise. Furthermore, the expression levels of *iNOS* were significantly decreased in the NA + Ex group as compared to the NA group. The expression levels of *Il-6* and *Il-1ra* did not significantly differ among the four groups. Additionally, in the comparison between the sedentary groups, application of jojoba oil caused no changes in the expression levels of the inflammation-related genes.

### 3.5. Effects of Exhaustive Exercise With or Without Topical Application of Jojoba Oil on the Expression Levels of Inflammation-Related Genes in the Heart

**Figure 5:**
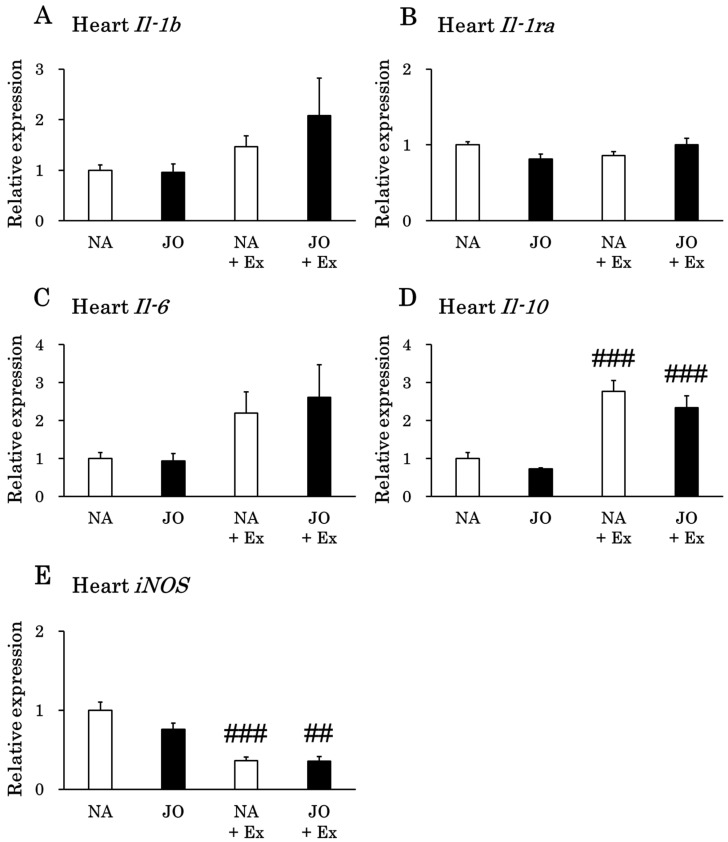
Gene expression levels of (**A**) *Il-1b*, (**B**) *Il-1ra*, (**C**) *Il-6*, (**D**) *Il-10*, and (**E**) *iNOS* in the heart. NA, the naïve control group; JO, the sedentary group treated with jojoba oil; NA + Ex, the exercise group without oil application; JO + Ex, the exercise group treated with jojoba oil. *Il-1b*, interleukin 1β; *Il-1ra*, interleukin 1 receptor antagonist; *Il-6*, interleukin 6; *Il-10*, interleukin 10; *iNOS*, inducible nitric oxide synthase. Values are presented as mean ± SE, *n* = 7. To compare the four groups, one-way analysis of variance was applied, followed by Tukey’s post hoc test. ## *p* < 0.01 as compared to the corresponding sedentary group; ### *p* < 0.001 as compared to the corresponding sedentary group.

Exhaustive exercise significantly elevated myocardial *Il-10* expression while reducing *iNOS* expression (Figure 5). In contrast, no significant differences were observed in the expression levels of *Il-1b*, *Il-1ra*, and *Il-6* across the four groups.

### 3.6. Effects of Exhaustive Exercise With or Without Topical Application of Jojoba Oil on the Expression Levels of Inflammation-Related Genes in the Liver

**Figure 6:**
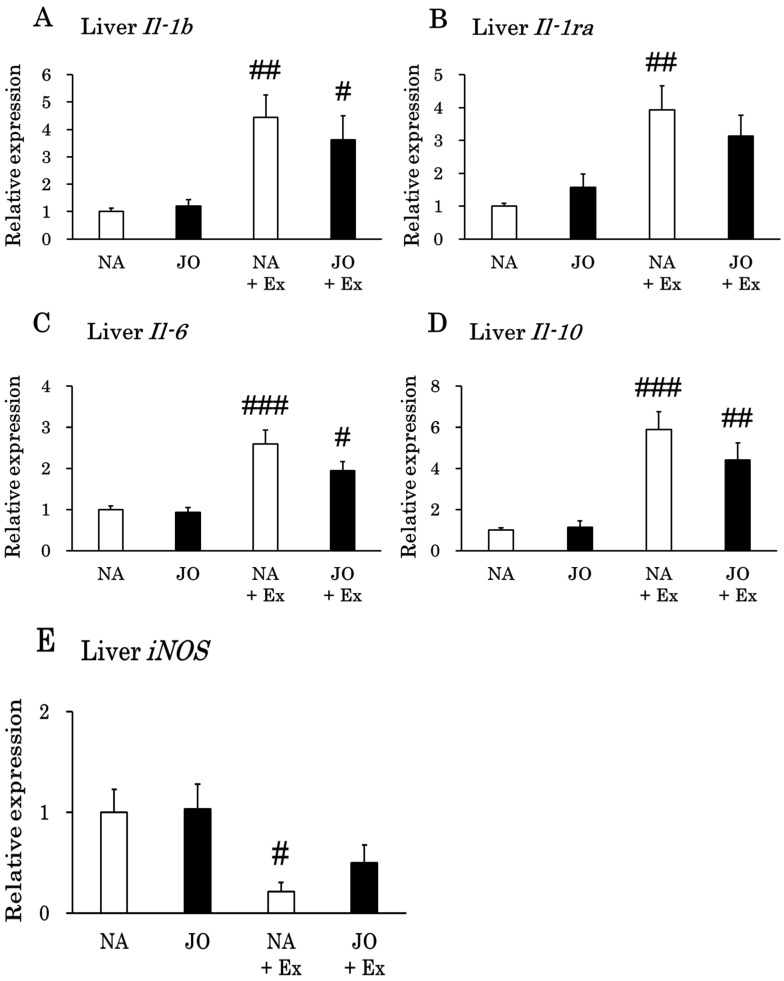
Gene expression levels of (**A**) *Il-1b*, (**B**) *Il-1ra*, (**C**) *Il-6*, (**D**) *Il-10*, and (**E**) *iNOS* in the liver. NA, the naïve control group; JO, the sedentary group treated with jojoba oil; NA + Ex, the exercise group without oil application; JO + Ex, the exercise group treated with jojoba oil. *Il-1b*, interleukin 1β; *Il-1ra*, interleukin 1 receptor antagonist; *Il-6*, interleukin 6; *Il-10*, interleukin 10; *iNOS*, inducible nitric oxide synthase. Values are presented as mean ± SE, *n* = 7. Data were analyzed by one-way ANOVA followed by Tukey’s post hoc test. # *p* < 0.05 as compared to the corresponding sedentary group; ## *p* < 0.01 as compared to the corresponding sedentary group; ### *p* < 0.001 as compared to the corresponding sedentary group.

Exhaustive exercise significantly increased the hepatic expression levels of *Il-1b*, *Il-6*, and *Il-10* (Figure 6). The expression level of the *Il-1ra* gene was also elevated by exercise, but only in the exercise group without oil application (NA + Ex). Conversely, the expression levels of *iNOS* were significantly decreased only in the NA + Ex group by exhaustive exercise.

### 3.7. Quantitative Analysis of Inflammation-Related Plasma Cytokine Levels

**Figure 7:**
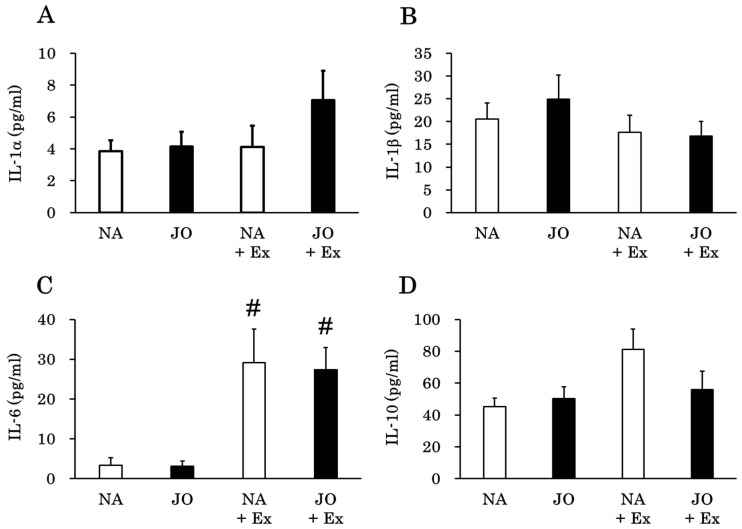

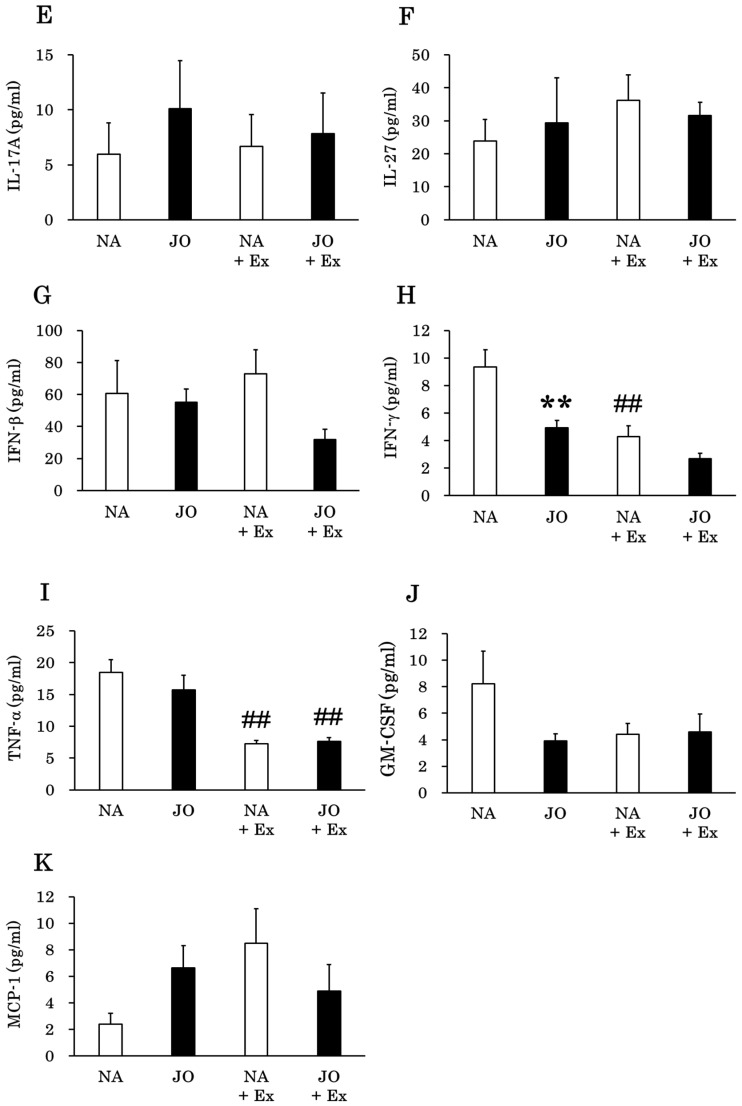
Plasma cytokine levels of (**A**) IL-1α, (**B**) IL-1β, (**C**) IL-6, (**D**) IL-10, (**E**) IL-17A, (**F**) IL-27, (**G**) IFN-β, (**H**) IFN-γ, (**I**) TNF-α, (**J**) GM-CSF, and (**K**) MCP-1 were determined by flow cytometry. NA, the naïve control group; JO, the sedentary group treated with jojoba oil; NA + Ex, the exercise group without oil application; JO + Ex, the exercise group treated with jojoba oil. IL, interleukin; IFN, interferon; TNF-α, tumor necrosis factor-α; GM-CSF, granulocyte-macrophage colony-stimulating factor; MCP-1, monocyte chemoattractant protein 1. Values are presented as mean ± SE, *n* = 7. Comparisons among groups were made using one-way ANOVA with Tukey’s post hoc test. ** *p* < 0.01 as compared to the NA group; # *p* < 0.05 as compared to the corresponding sedentary group; ## *p* < 0.01 as compared to the corresponding sedentary group.

In the exercise groups, the application of jojoba oil was found to reduce the gene expression levels of inflammatory cytokines, especially in the soleus muscle. To confirm whether systemic cytokines were altered in response to the topical application of jojoba oil, plasma cytokine levels were quantified by flow cytometry, as presented in Figure 7. The plasma levels of IL-1α, IL-1β, IL-10, IL-17A, IL-27, IFN-β, GM-CSF, and MCP-1 were not significantly different among the four groups. In contrast, while plasma levels of IL-6 were significantly elevated by exhaustive exercise in the NA + Ex and JO + Ex groups, those of TNF-α were significantly decreased in these same groups. Additionally, with respect to the sedentary groups, the plasma levels of IFN-γ were significantly decreased in the JO group as compared to those in the NA and significantly decreased in the NA + Ex group compared to those in the NA group. However, within the exercise groups, there were no significant changes between the NA + Ex and JO + Ex groups for all cytokines measured, and the plasma levels of IL-12p70 and IL-23 were below the limit of detection (2.54 pg/mL and 33.09 pg/mL, respectively).

### 3.8. Correlation Between Plasma CK Levels and Time to Exhaustion With or Without Topical Application of Jojoba Oil

**Figure 8:**
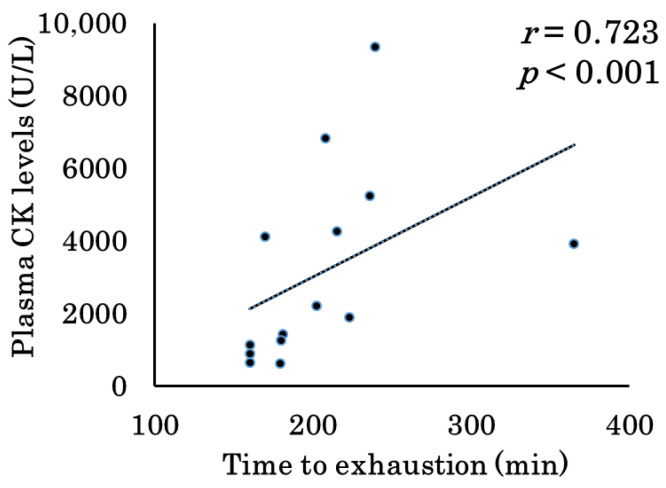
Correlation between plasma creatine kinase (CK) levels and time to exhaustion in the exercise groups with or without topical application of jojoba oil (NA + Ex and JO + Ex). Plasma CK levels were significantly correlated with time to exhaustion (*r* = 0.723, *p* < 0.001). Correlations were examined using Spearman’s rank correlation analysis.

To investigate whether prolonged exercise duration is related to muscle injury, we evaluated the correlation between plasma CK levels, a reliable marker of muscle injury, and time to exhaustion (Figure 8). Spearman’s rank correlation analysis showed a significant positive correlation between plasma CK levels and the time to exhaustion (*r* = 0.723, *p* < 0.001).

### 3.9. Correlation Between Plasma CK Levels and Inflammation-Related Genes Expressed in Skeletal Muscles with and Without Topical Application of Jojoba Oil

**Figure 9:**
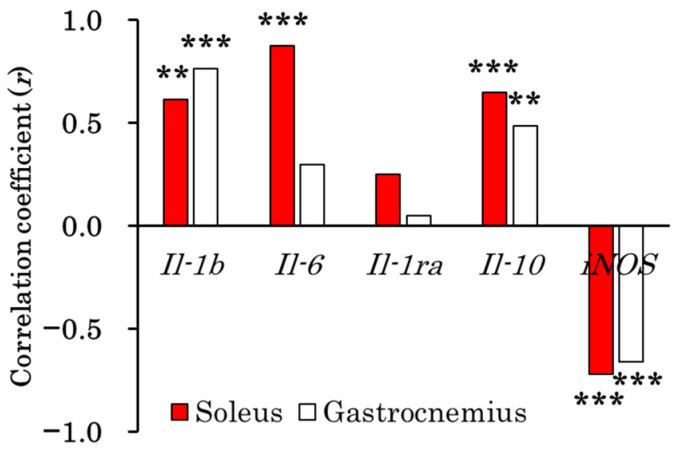
Correlation between plasma creatine kinase (CK) levels and inflammation-related genes expressed in soleus (slow-twitch muscle) and gastrocnemius muscles (fast-twitch muscle) with and without topical application of jojoba oil (NA + Ex and JO + Ex). Plasma CK levels showed a significant correlation with the mRNA expression of several genes: *Il-1b*, *Il-6*, *Il-10*, and *iNOS* in the soleus muscle, and *Il-1b*, *Il-10*, and *iNOS* in the gastrocnemius muscle. However, no significant correlation was observed between plasma CK levels and *Il-1ra* expression levels in either muscle. Correlations were examined using Spearman’s rank correlation analysis. ** *p* < 0.01; *** *p* < 0.001.

As a significant correlation was observed between treadmill running time and plasma CK levels (Figure 8), we investigated the relationship between plasma CK levels and the expression levels of pro-inflammatory cytokines/enzymes (*Il-1b*, *Il-6*, and *iNOS*) and anti-inflammatory cytokines (*Il-1ra* and *Il-10*) in the soleus and gastrocnemius muscles, combining the groups with and without topical jojoba oil application. As shown in Figure 9, plasma CK levels were significantly correlated with *Il-1b* (*r* = 0.617, *p* < 0.01), *Il-6* (*r* = 0.874, *p* < 0.001), *Il-10* (*r* = 0.650, *p* < 0.001), and *iNOS* (*r* = −0.720, *p* < 0.001) expression in the soleus muscle. In the gastrocnemius muscle, plasma CK levels were significantly correlated with *Il-1b* (*r* = 0.764, *p* < 0.001), *Il-10* (*r* = 0.487, *p* < 0.01), and *iNOS* (*r* = −0.660, *p* < 0.001) expression.

## 4. Discussion

### 4.1. Effect of Topical Application of Undiluted Jojoba Oil Before Exercise on Endurance Performance and Plasma Biochemical Parameters

The present study aimed to determine whether topical application of undiluted jojoba oil prior to treadmill running could prevent exercise-induced muscle injury and influence the time to exhaustion. A recent study reported that plasma NEFA levels increased 30 min after transdermal administration of jojoba oil in mice [25]. Based on these findings, we examined whether applying jojoba oil 30 min before exercise would prolong the time to exhaustion during treadmill running. Our results demonstrated that jojoba oil application did not influence endurance capacity (Figure 1). This outcome can be interpreted considering the plasma biochemical data (Figure 2). In the exercise groups (NA + Ex and JO + Ex), plasma GLU and LA concentrations were lower than in the sedentary groups (NA and JO), suggesting their utilization as energy sources. However, there were no significant differences in plasma GLU and LA levels between the NA + Ex and JO + Ex groups. Plasma IL-6 levels were elevated in both exercise groups compared with sedentary controls, yet there was no significant difference between the two exercise groups. Although IL-6 is widely recognized as a pro-inflammatory cytokine, previous studies have reported that physiological concentrations can also exert anti-inflammatory effects [31] and enhance metabolism [24,32]. In the present study, the absence of a difference in time to exhaustion between the NA + Ex and JO + Ex groups suggests there was no differential effect of IL-6 on energy metabolism. From a practical standpoint, given that topical application of jojoba oil did not improve endurance performance, it may be used for sports massage without concern regarding doping regulations.

Furthermore, CK, a well-established marker of muscle injury [33], along with ALT, AST, and ALP, which are markers of liver injury [34,35], and BUN and CRE, which serve as markers of renal injury, were all elevated in both the NA + Ex and JO + Ex groups. This confirms that the treadmill protocol was sufficiently intense to induce physiological stress. The absence of significant differences in these markers between the two exercise groups indicates that topical application of jojoba oil prior to treadmill running does not mitigate reversible exercise-induced damage to the liver, kidneys, or skeletal muscles.

Interestingly, plasma NEFA levels were significantly lower in the JO group compared with the NA group (Figure 2). This finding contrasts with previous work [25], possibly because the interval between oil application and blood sampling differed between the studies (30 min in the previous report vs. approximately 3 h in the present study). In our experiment, blood was collected approximately 3 h after oil application to match the sampling time of the corresponding exercise groups. Additionally, discrepancies between the two studies may be attributable to circadian variations in lipid metabolism [36,37].

### 4.2. Effects of Exhaustive Exercise With or Without Topical Application of Jojoba Oil on the Expression Levels of Inflammation-Related Genes in Various Tissues/Organs

The aim of the present study was to investigate whether pre-exercise topical application of jojoba oil mitigates exercise-induced inflammation. To address this question, we quantified the expression of inflammation-related mRNAs in the soleus muscle, gastrocnemius muscle, heart (cardiac muscle), and liver in mice after jojoba oil application prior to treadmill running. The expression levels of *Il-1b* and *Il-6* (pro-inflammatory cytokines) as well as *Il-1ra* and *Il-10* (anti-inflammatory cytokines) were evaluated. IL-1ra is known to inhibit IL-1β signal transduction [38], while IL-10 suppresses the synthesis of inflammatory cytokines, such as TNF-α [39]. Therefore, we analyzed *Il-1ra* and *Il-10* expression as representative anti-inflammatory markers. In addition, because iNOS is upregulated in inflamed tissues by inflammatory cytokines and contributes to cytotoxicity, the expression levels of *iNOS* were also evaluated. iNOS is widely recognized as a pro-inflammatory enzyme because it generates NO at levels 20–50 times higher than those produced by the constitutive isoforms of NOS [40]. The present study demonstrated that exhaustive exercise significantly increased *Il-1b* and *Il-6* expression in the soleus muscle and liver. Similarly, in the gastrocnemius muscle, *Il-1b* expression was significantly elevated following exhaustive exercise. In contrast, no significant increases in the expression of *Il-1b* or *Il-6* were detected in the heart following exhaustive exercise.

We next examined whether jojoba oil, which possesses anti-inflammatory properties, could attenuate the exercise-induced upregulation of these cytokines. Notably, pre-exercise jojoba oil application significantly suppressed *Il-1b* and *Il-6* expression in the soleus muscle and *Il-1b* expression in the gastrocnemius muscle after exhaustive exercise (Figure 3 and Figure 4). These findings suggest that topical jojoba oil applied before exercise may reduce inflammation in lower-limb skeletal muscles. In contrast, jojoba oil did not attenuate the exercise-induced increases in liver *Il-1b* and *Il-6* expression. In all tissues/organs examined, *iNOS* expression was significantly reduced after exhaustive exercise. The topical application of jojoba oil did not alter *iNOS* expression when comparing the NA + Ex and JO + Ex groups. Interestingly, previous reports have yielded contrasting results: five consecutive days of treadmill running significantly increased *iNOS* expression [41], whereas gastrocnemius muscle *iNOS* expression was significantly reduced 2 h after swimming in high-fat diet-fed mice [42]. Taken together, these observations suggest that a single bout of exercise decreases *iNOS* expression, whereas repeated forced exercise without adequate muscle recovery leads to its upregulation.

To explore a potential mechanism for the anti-inflammatory effects of jojoba oil, we hypothesized that reduced pro-inflammatory cytokine expression might be accompanied by an increase in anti-inflammatory cytokine expression. We therefore analyzed *Il-1ra* and *Il-10* expression levels in view of the cytokine network regulation following exercise [24,43]. Exhaustive exercise significantly increased *Il-10* expression in the soleus muscle, heart, and liver, whereas *Il-1ra* expression was significantly elevated only in the liver. However, comparison of the NA + Ex and JO + Ex groups revealed that the topical administration of jojoba oil did not enhance hepatic anti-inflammatory cytokine gene expression following exercise. Interestingly, *Il-10* expression in the gastrocnemius muscle was significantly increased only in the JO + Ex group following exercise, suggesting that jojoba oil may upregulate *Il-10* expression specifically in this muscle. Conversely, soleus *Il-10* expression was significantly lower in the JO + Ex group than in the NA + Ex group, a change that may reflect the concomitant reduction in soleus *Il-6* expression observed in the JO + Ex group [24,31,43].

Research on the anti-inflammatory effects of jojoba oil suggests a multi-faceted mechanism of action. According to a prior study [23], jojoba oil and its formulations reduce inflammation by altering key cellular and molecular signals. In an ex vivo study using human skin, jojoba wax was shown to lower IL-6 and IL-8 by approximately 30% and to reduce TNF-α to levels comparable to those achieved with dexamethasone [44]. Similarly, in in vivo rat models of lung injury and skin lesions, jojoba nanoemulsions decreased inflammatory cytokines such as IL-1β, IL-6, and TNF-α. They also reduced reactive oxygen species and malondialdehyde, suppressed neutrophil infiltration and myeloperoxidase activity, and increased the anti-inflammatory cytokine IL-10 [45]. Other research has also noted similar effects, such as reduced prostaglandin E2 and nitric oxide, and improved histopathology in models of paw and dental pulp inflammation [23]. Collectively, these findings indicate that the anti-inflammatory action of jojoba oil arises from a coordinated modulation of pro- and anti-inflammatory cytokines, a decrease in oxidative stress, and an attenuation of inflammatory cell recruitment and tissue edema [45].

The molecular mechanisms behind jojoba oil’s anti-inflammatory properties are also being uncovered. Jojoba oil contains bioactive glycosides that reduce inflammation [46]. Specifically, the non-cyanogenic cyanoglucoside dimers, simmonosides A and B, directly inhibit cyclooxygenase 2 in vitro with half-maximal inhibitory concentrations of 13.5 and 11.4 μM, respectively [47]. In parallel, the cyanogenic glycoside simmondsin from jojoba seeds has been shown to lower reactive oxygen species by up to 69% and modulate caspase 3 activity [46]. In formulations such as nanoemulsions enriched with gallic acid, suppression of nuclear factor κB and its downstream cytokines is also observed [45]. These findings suggest that specific glycoside compounds in jojoba oil exert their anti-inflammatory effects through the inhibition of cyclooxygenase 2 and the reduction in oxidative stress [46].

### 4.3. Quantitative Analysis of Inflammation-Related Plasma Cytokine Levels by Flow Cytometry

Flow cytometric analysis [24] revealed that IL-6 was the only inflammatory cytokine found to be elevated by exercise among those measured in this study, and no reduction in its plasma concentration due to topical jojoba oil administration was observed. Since IL-6 is a myokine secreted in response to muscle contraction [24,43,48], the soleus muscle likely contributed to the increase in plasma IL-6 levels during exhaustive exercise. Additionally, since cytokines are produced by various tissues and immune cells [24,43,49], other tissue sources may also have contributed to the increase in plasma levels of IL-6. For example, shear stress increases as blood flow to skeletal muscle is enhanced during exercise [50], and shear stress on bovine endothelial cell monolayers has been shown to induce IL-1β and IL-6 secretion [51].

The plasma concentration of IL-1β was not increased by exercise; however, exercise elevated *Il-1b* mRNA expression in both the soleus and gastrocnemius muscles. Topical jojoba oil significantly attenuated the expression of these exercise-induced inflammatory cytokines, suggesting that jojoba oil may suppress exercise-induced local inflammation in skeletal muscle. Previous studies have also reported that exercise increases plasma IL-6 levels [24,43,52], and the present findings are consistent with those reports. While plasma inflammatory cytokine levels also rise during endotoxemia, such as sepsis, it has been reported that TNF-α and IL-1β do not increase in response to exercise-induced cytokine production [24,43,53], a pattern similarly observed in this study. The post-exercise elevation of IL-6 likely reflects not only inflammation, but also its role in promoting intracellular glucose uptake and lipid metabolism [24,54,55]. Therefore, among the inflammatory cytokines measured here, plasma IL-6 levels may be particularly sensitive to exercise.

In the sedentary condition, plasma IFN-γ levels were lower in the JO group compared with the NA group. Further research is warranted to elucidate the underlying mechanisms. IFN-γ, a cytokine secreted primarily by T cells and natural killer cells, acts to enhance inflammation [24,43]; this reduction may reflect an anti-inflammatory effect of jojoba oil. Given that exhaustive exercise is known to transiently suppress immune function [24,43,56,57], the lower plasma IFN-γ levels in the NA + Ex group compared with the NA group are likely due to exercise-induced reductions in lymphocyte function. Mechanistically, synthetic glucocorticoids have been shown to inhibit IFN production [58,59]. Moreover, exercise-induced increases in plasma cortisol and epinephrine may contribute to the suppression of type 1 helper T-cell cytokine (IFN-γ) production [24]. Similarly, the observed decrease in plasma TNF-α in the exercise groups may be attributable to epinephrine [43,60,61] and IL-6 [24,52,62,63] release during exercise, both of which are known to suppress TNF-α production.

### 4.4. Correlation Between Plasma CK Levels and Inflammation-Related Gene Expression in Skeletal Muscle With or Without Topical Application of Jojoba Oil

Exhaustive exercise induces muscle injury. In this study, the biochemical parameters measured included CK and AST, both of which are abundant in skeletal muscle and serve as reliable markers of muscle injury. ALT, although present in smaller amounts in skeletal muscle, can also be released into the bloodstream during muscle injury. The plasma concentrations of these markers increased significantly following exercise (Figure 2). Plasma CK levels have been widely used in numerous studies as an index of muscle injury [33]. In contrast, plasma AST and ALT are also markers of liver injury and lack specificity for skeletal muscle. Therefore, we analyzed the correlation between plasma CK level—as a marker of muscle injury—and running time using Spearman’s rank correlation, confirming that CK is a reliable muscle injury marker in this experimental system (Figure 8). We then examined the relationship between muscle injury (elevated plasma CK) and the expression of inflammation-related cytokine genes in skeletal muscle (Figure 9). Plasma CK levels were positively correlated with *Il-1b* and *Il-6* expression in the soleus muscle, and with *Il-1b* in the gastrocnemius muscle. These findings are consistent with results showing that topical jojoba oil attenuated the exercise-induced upregulation of inflammatory cytokine genes in muscle (Figure 3 and Figure 4). Collectively, these data suggest that jojoba oil may suppress the expression of genes that are closely associated with exercise-induced muscle injury.

A correlation was also observed between plasma CK levels and *Il-10* expression in both the soleus and gastrocnemius muscles, potentially reflecting the induction of plasma IL-10 via elevated IL-6 concentrations [24,31,43,62,63]. Notably, in the gastrocnemius muscle, *Il-1b* was the only gene correlated with plasma CK levels, whereas in the soleus muscle, both *Il-6* and *Il-1b* exhibited significant correlations. This pattern may indicate the greater involvement of slow-twitch fibers in the endurance treadmill protocol employed in this study.

Interestingly, a negative correlation was identified between elevated plasma CK levels and *iNOS* expression in skeletal muscle, which may reflect the marked post-exercise reduction in *iNOS* expression observed in this study.

### 4.5. Jojoba Oil as a Novel Therapeutic Strategy for Exercise-Induced Muscle Injury

Therapeutic strategies for exercise-induced muscle injury typically include stretching, massage, nonsteroidal anti-inflammatory drugs (NSAIDs), and nutritional support [7,8,11,16]. While NSAIDs are widely prescribed in clinical practice, they are associated with adverse effects such as gastrointestinal bleeding [64,65]. Given these limitations, we propose jojoba oil as a novel and promising therapeutic candidate, capitalizing on its favorable safety profile and unique biological properties.

Jojoba oil has an established record of safety, supported by its long-standing topical use as both a moisturizer in skincare and a lubricant in sports massage. Its remarkable compatibility with human skin, attributable to its structural similarity to the wax esters present in human sebum [66], has contributed significantly to this safety profile. Transdermal delivery offers several advantages over other routes of administration, including the ability to bypass the first-pass effect before entering systemic circulation [67,68,69]. Moreover, this route enables gradual absorption through the skin, thereby providing sustained therapeutic effects [70], maintaining a stable and prolonged systemic concentration [68,69], and offering a simple, non-invasive method of administration [68,69]. In addition, massage itself has been reported to exert anti-inflammatory effects [71]. Thus, sports massage with jojoba oil may yield a synergistic anti-inflammatory benefit, surpassing that of either jojoba oil or massage alone. Further human studies are warranted to elucidate and confirm this potential synergistic effect.

### 4.6. Study Limitations and Future Perspectives

Our study has several limitations. A limitation of this study is the lack of a detailed chemical profile of the trace components of the jojoba oil used. The most comprehensive data available were limited to the analysis certificate from the manufacturer’s accredited institution, which identified the three major fatty acids (eicosenoic acid, erucic acid, and oleic acid) but did not analyze the remaining trace components. Another limitation is that we did not systematically investigate the optimal timing and volume for jojoba oil application. While we chose the 30 min pre-exercise timing and a specific volume based on previous pharmacokinetic research [25,27] and practical considerations, our findings cannot definitively determine the most effective application window or quantity. Future research should systematically compare different application timings (e.g., immediately, 30 min, or 60 min before exercise), volumes, and durations. Furthermore, the use of an animal model presents an additional limitation. Although rodents are widely employed to investigate exercise-induced muscle injury, differences in skin morphology between animals and humans may influence the transdermal absorption of jojoba oil. Variations in the density of hair follicles, sweat glands, and overall stratum corneum thickness could affect penetration efficiency and, consequently, the observed physiological effects. Therefore, caution is warranted when extrapolating the present findings to humans, and further studies in applied sports settings are required.

While this study demonstrated the potential efficacy of jojoba oil, further investigation is required to establish its practical application in sports and exercise settings. Future studies should aim to evaluate the anti-inflammatory and antioxidant effects of jojoba oil in various organs such as the lungs, liver, and kidneys following exhaustive exercise [72]. This is crucial for a comprehensive understanding of how jojoba oil affects systemic inflammatory responses. Furthermore, to elucidate the anti-inflammatory mechanisms of jojoba oil suggested by this study, it is necessary to identify which component is active and clarify the involved signal transduction pathways using cellular experiments and gene expression analysis. Additionally, investigating the effectiveness of jojoba oil against other types of muscle injuries, such as contusions and sprains, would broaden its potential applications. Another important area for future research is to examine how jojoba oil influences the function and strength of active muscles during exercise, which is a significant consideration for its application in sports settings. Collectively, these efforts could pave the way for establishing jojoba oil as a valuable supportive strategy for enhancing recovery and reducing exercise-induced muscle inflammation.

## 5. Conclusions

This study demonstrated that topical jojoba oil application administered 30 min prior to treadmill running did not affect the endurance capacity of mice. It also had no significant effect on plasma cytokine levels in mice subjected to exhaustive exercise. However, the topical application of jojoba oil markedly reduced the mRNA expression of specific pro-inflammatory cytokines (IL-1β and IL-6), which are typically upregulated after exhaustive exercise—most notably in the soleus muscle, a slow-twitch muscle essential for endurance exercise. Taken together, these findings indicate that pre-exercise topical application of jojoba oil has the potential to attenuate exercise-induced skeletal muscle inflammation. Therefore, our results suggest that sports massage incorporating jojoba oil may offer a practical strategy to mitigate exercise-induced muscle injury and inflammation.

## Figures and Tables

**Table 1:** Fatty acid composition of jojoba oil.

Name	MW [g/moL]	Content [%]
Eicosenoic acid	310.51	73.4
Erucic acid	338.57	14.7
Oleic acid	282.47	8.3

MW, molecular weight.

**Table 2:** Primers for real-time PCR analysis.

Gene	Accession No.	Forward	Reverse
*Il-1b*	NM_008361.4	TGCCACCTTTTGACAGTGATG	TGTGCTGCTGCGAGATTTGA
*Il-1ra*	NM_031167.5	TGTGCCAAGTCTGGAGATGA	TTCTTTGTTCTTGCTCAGATCAGT
*Il-6*	NM_001314054.1	GCTACCAAACTGGATATAATCAGGA	CCAGGTAGCTATGGTACTCCAGAA
*Il-10*	NM_010548.2	CAGAGCCACATGCTCCTAGA	TGTCCAGCTGGTCCTTTGTT
*iNOS*	NM_001313922.1	GGGCTGTCACGGAGATCA	CCATGATGGTCACATTCTGC
*Actb*	NM_007393.5	CCTCCCTGGAGAAGAGCTATG	TTACGGATGTCAACGTCACAC

*Il-1b*, interleukin 1β; *Il-1ra*, interleukin 1 receptor antagonist; *Il-6*, interleukin 6; *Il-10*, interleukin 10; *iNOS*, inducible nitric oxide synthase; *Actb*, β-actin.

## Data Availability

Data are available from the corresponding author on request.
